# Endodontic Treatment of Complex Dens Invaginatus Teeth with Long Term Follow-Up Periods

**DOI:** 10.22037/iej.v13i2.19302

**Published:** 2018

**Authors:** Natália Gomes de Oliveira, Marina Torreão da Silveira, Shirley Machado Batista, Sirley Raiane Mamede Veloso, Marianne de Vasconcelos Carvalho, Rosana Maria Coelho Travassos

**Affiliations:** a *Department of Operative Dentistry and Endodontics, Dental College of Pernambuco, University of Pernambuco, Camaragibe-PE, Brazil*

**Keywords:** Anomalies, Classification, Dens Invaginatus, Root Canal Therapy

## Abstract

*Dens invaginatus* is characterized by invagination of enamel and dentin in the dental papilla prior to tissue calcification. This malformation commonly occurs in the maxillary lateral incisors. The present study reports two complex endodontic treatments in Oehlers’ type II and III *dens invaginatus,* with periapical lesion and presence of bone resorption. In the reported cases, conventional endodontic therapy was successful and sufficient enough to eliminate the infectious process, allowing periapical bone neoformation and absence of symptomatology. *Dens invaginatus* is a relatively easy-to-diagnose dental malformation. However, it is necessary to know its radiographic aspects. The treatment results demonstrated that, although the cases of *dens invaginatus* of high complexity are challenging, an accurate diagnosis accompanied with proper endodontic treatment can avoid unnecessary surgical intervention and allow great chances of favorable prognosis in long term.

## Introduction

Dens invaginatus is characterized by invagination of enamel and dentin in the dental papilla prior to tissue calcification [1]. It is a malformation that commonly occurs in the maxillary lateral incisors. Such invagination may be restricted only to the pulp chamber, the root or in more extreme cases, it may even reach the apex [2, 3]. The incidence has been reported to vary from 0.04% to 10%. There is no consensus regarding its etiology, and several theories have been proposed, the most plausible being a retardation or stimulation of focal growth in certain areas of the dental germ, also inadequate bone development and its consequent enamel arch constriction, external trauma, infections, nutritional and genetic factors, with the latter being the most likely. It is possible that more than one factor contributes to the occurrence of the malformation [4].

Although there are some classification systems, the most commonly used is Oehlers’. According to this classification, based on the extent of invaginated dental tissues and the communication with the periapical or periodontal, there are 3 types of invagination [5]. Type I shows an invagination limited to the crown. Type II extends below cemento-enamel junction, ends as a blind sac, and may or may not have communication with the pulp. Type III extends through the root and perforates the apical or lateral region, without any immediate communication with the dental pulp [6].

Diagnosis is based on clinical and radiographic examination. The clinical appearance varies considerably and may appear normal or associated with unusual forms, such as greater buccolingual dimension, in the shape of a shell or cylinder and in conical shapes [7]. Radiographs represent the main diagnostic resource, with an image that can appear as one tooth inside the other.

**Figure 1:**
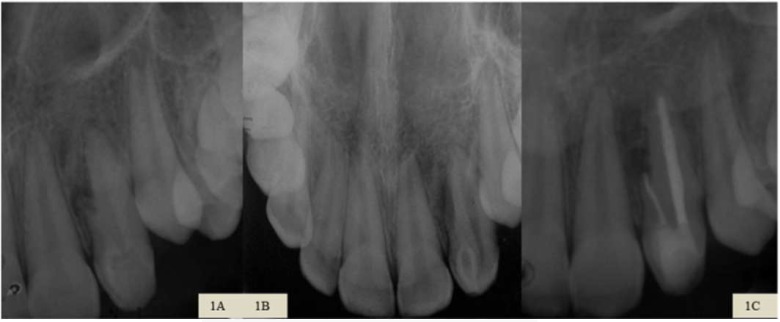
*A)* Initial periapical radiography; *B)* Initial occlusal radiograph for verification of dens invaginatus in tooth 22 associated with lateral bone resorption; *C)* Final periapical radiography with root canal filling and restoration in tooth 22

The main consequence of dens invaginatus is the largest accumulation of debris, which can end in a rapid onset and progression of dental caries, with subsequent involvement of the pulp, resulting in pulp/periapical pathology.

After pulp/periapical involvement, depending on the degree of complexity and classification of invagination, several treatment modalities have been prescribed for these teeth. Options include conservative endodontic treatment, endodontic surgery, intentional re-implantation and tooth extraction [8].

The objective of the present study is to report two complex endodontic treatments, in Oehlers’ type II and III dens invaginatus classification; with periapical lesion and presence of bone resorption.

## Case Report

***Case 1***

A 13-year-old female patient was referred to the endodontic clinic of Centro de Pós Graduação em Odontologia - CPO (Post-Graduation Center in Dentistry) in Recife, PE, Brazil, for endodontic evaluation of tooth 22. During the anamnesis, trauma was reported 3 years earlier. The patient reported that she had undergone parendodontic surgery 6 months earlier and a fistula appeared 1 month after the treatment. Antibiotic medication was prescribed, but according to the patient, fistula episodes were recurrent. The physical examination revealed an active fistula in the vestibular region of tooth 22, pain at vertical percussion and palpation, and absence of symptomatology to the cold thermal stimulus (Endo-ice, Hygenic Corp., Akron, OH, USA). From the periapical and occlusal radiography, we verified the presence of dens invaginatus associated with a lateral bone resorption (Figures 1A and 1B). Fistula tracking was performed and involvement of the evaluated tooth was confirmed. Chronic apical abscess was diagnosed therefore; a conservative endodontic treatment was opted for.

**Figure 2:**
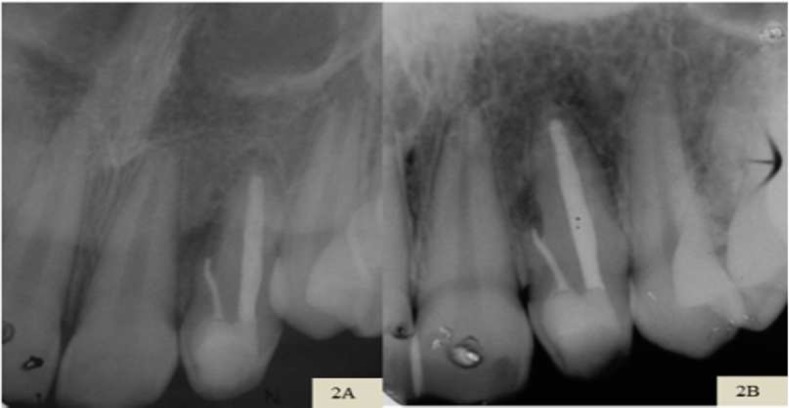
*A)* Periapical follow-up radiography after 1 year of endodontic treatment; demonstrating a decrease in periapical lesion; *B)* After 3 years; exhibiting bone neoformation

In the first session of the treatment, and before the access opening procedure, prophylaxis was performed with pumice stone and water, aiming at reducing bacterial contamination. Then, coronary opening was performed to have appropriate access to the main and invaginated canals. During the opening phase, pulp vitality was visually confirmed in the main canal and necrosis was seen in the dens invaginatus canal. The vital main canal presented characteristics of a vital pulp with bleeding while the necrotic invaginated canal showed no bleeding. In both canals, total length measurement and biomechanical preparation were performed by the manual crown-down technique with K-files (Maillefer, Ballaigues, Switzerland). The irrigating solution used was 2.5% sodium hypochlorite and a series of different instruments were carefully used in the radicular spaces. In order to avoid infection, Calcium hydroxide paste was applied (Calen, SS White, Artigos Dentarios Ltda., Rio de Janeiro, Brazil) and the tooth was sealed with a temporary glass ionomer cement restoration (Vitro Fill, DFL, Brazil). 

At one-week follow-up appointment, the patient returned pain free and the obturation of the root canal system was performed. The endodontic cement used was Sealer 26 (Dentsply Maillefer, Petrópolis, RJ, Brazil) and lateral condensation was used as the obturation technique. The final restoration was done with Z-100 composite resin (3M ESPE, St. Paul, MN, USA) (Figure 1C).

In the radiographic follow-up after 14 months, the size of the periapical lesion was reduced and, 3 years and 7 months after the treatment, new bone formation was observed (Figures 2A and 2B). Clinically the patient presented an aspect of normality, with absence of symptoms.

***Case 2***

A 19-year-old female patient was referred to the Endodontic Clinic of Centro de Pós Graduação em Odontologia -CPO (Post-Graduation Center in Dentistry) in Recife, PE, Brazil, for an endodontic evaluation of tooth 12. The patient reported that the previous professional indicated parendodontic surgery. During the anamnesis, the patient reported trauma in this region - which occurred 11 years earlier - a recurrent increase in volume in the hard palate, beside localized and provoked pain in the related tooth. 

**Figure 3:**
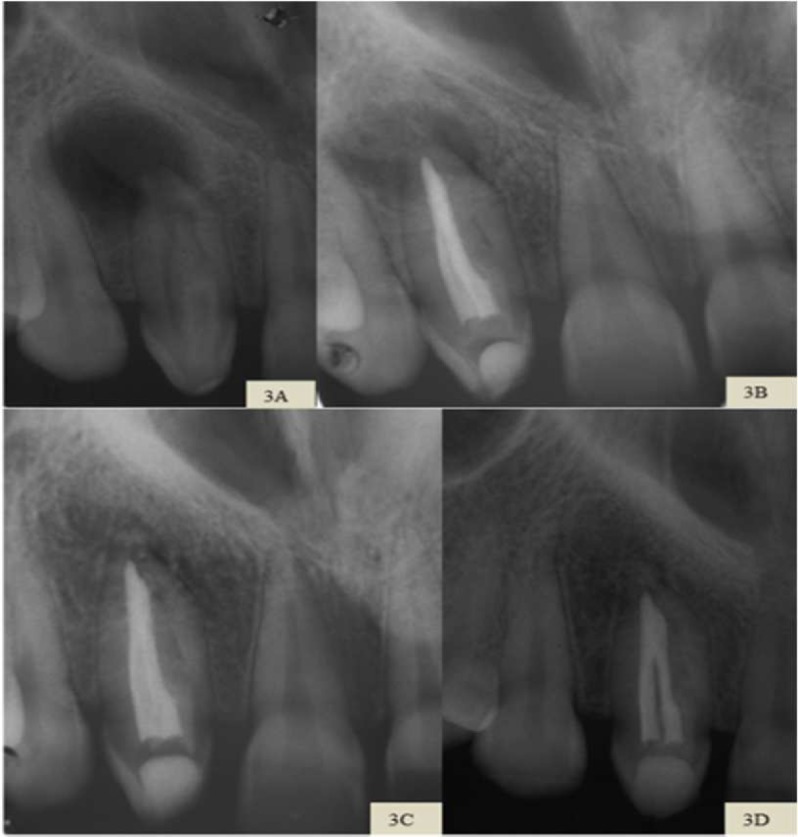
*A)* Initial periapical radiography showing bone resorption and presence of invagination in tooth 12; *B)* Final periapical radiography with root canal filling and restoration in tooth 12; *C)* Periapical follow-up radiography after 1 year and 9 months with reduction of periapical lesion and verification of bone neoformation; *D)* After 3 years; demonstrating the normality of the periapical tissues

Physical examination revealed active fistula on the palate, pain on vertical percussion and palpation in the referred region, also absence of symptomatology for the thermal stimulation. Radiographic examination revealed circumscribed bone resorption and the presence of dens invaginatus in tooth 12 (Figure 3A). Fistula tracking was performed and involvement of the related tooth was confirmed. A chronic apical abscess was diagnosed and conservative endodontic treatment was indicated.

In the first session of the treatment, prophylaxis was performed with pumice stone and water, before the coronary opening procedure. Then, coronary opening was conducted for proper access to the main and the dens invaginatus canals. During the exploration of the canals, it was difficult to reach the working length in the dens invaginatus canal due to a calcified barrier in the middle third. Then, total length measurement and biomechanical preparation of the main canal were performed by a manual crown-down technique with K-files (Maillefer, Ballaigues, Switzerland). Calcium hydroxide paste was applied in the two canals and the tooth was sealed with a temporary glass ionomer cement restoration. Systemic medication was prescribed: Clavulin (GSK) 500 mg every 8 h, for seven days and Celebra (Pfizer) 200 mg per day for five days. The antibiotic prescription was chosen because of the difficulty during the patency in the canal, preventing an adequate chemo-mechanical preparation. Another factor contributing to this decision was the recurrence of volume increase in the palatine region of the tooth. At one-week follow-up appointment, the patient returned pain free and the dens invaginatus canal was accessed to try to reach the working length. As the total length of the canal was estimated to be 18 mm and only 10 mm penetration was possible, a diamond spherical tip (Irrisonic; Helse Dental Technology, Santa Rosa do Viterbo, Brazil) was used to try to find the canal continuity. A radiographic image was taken shortly afterwards and a communication with the main channel was observed. Then, the crown-down technique was performed and calcium hydroxide paste were prepared as the intracanal medication, completely filling the root canal system. The medication remained for a period of 60 days.

In the third session, the patient reported mild onset of localized edema in the palate, with no painful symptoms. The intracanal medication was renewed for another 30 days. The patient only returned for the conclusion of the treatment 3 months later, without any symptoms and absence of edema. The obturation of the canals was performed using the Tagger hybrid technique and Sealapex (Kerr Corporation, Orange, CA, USA). The restoration was performed with composite resin Z100 (3M ESPE, St. Paul, MN, USA) (Figure 3B).

In the follow-up radiography, after 21 months, a decrease in the size of the periapical lesion was observed, with bone neoformation (Figure 3C) and, currently, after 3 years, the radiographic appearance of the periapical tissues was normal (Figure 3D). Clinically, the patient did not report any symptoms.

## Discussion

In the absence of significant clinical signs or symptoms, teeth with dens invaginatus, may go unnoticed on a routine clinical examination. Its recognition usually occurs when the patient looks for a professional care as a consequence of clinical characteristics of pulp/periapical pathology, such as the presence of a fistula related to the affected tooth [9]. This fact was observed in the two clinical cases of the present report, in which the diagnosis was prompted due to the appearance of a fistula associated with pain on vertical percussion and the palpation and also the negative response to the cold thermal stimulus.

Radiography, usually periapical or occlusal, is the best way to diagnose dens-Invaginatus [10]. In both cases, these examinations revealed the appearance of one tooth within* the other*, coming to the diagnosis of dens invaginatus according to Oehlers’ classification. The first case was classified as type III, characterized with invagination extending throughout the root perforating the apical or extra lateral foramen. The second case was classified as type II, represented by its extension below the cement-enamel junction, with pulp communication [5]. The radiographs revealed bone rarefaction as a result of the evolution of the infectious process.

Accurate diagnosis of dens invaginatus is essential to decide the type of treatment. If the teeth are healthy, it is crucial to give proper oral hygiene instructions since a greater possibility of accumulation of debris in the involved tooth can increase the risk of tooth decay. If caries is present, restorations should be considered next to hygiene guidance and follow-up in order to avoid microleakage. When there is pulp/periapical infection, endodontic therapy should be the first choice.

In the reported clinical cases, the initial suggested treatment plans of the previous professionals were parendodontic surgery, demonstrating the limited awareness regarding the diagnosis and treatment of this type of malformation. When they were referred to the endodontist, the precise diagnosis was made and the necessary endodontic treatments were performed for both patients. These treatments had excellent long-term results, as it aimed to disinfect the entire root canal system, rather than just apical sealing, a therapy which is proposed in some surgical procedures [11].

In the case of pulp/periapical involvement, the complex anatomy of teeth with dens invaginatus, especially type II and III cases is a challenge during an endodontic treatment. Such complexity often makes it difficult or even impossible to adequately disinfect and obturate the root canal system. Amongst the clinical cases presented, the mechanical difficulties were greater in case report II, due to the more extreme anatomical complexity of the tooth in question; therefore, it was necessary to use an ultrasound tip in the middle third to access the canal of the dens invaginatus. 

In the first case, although the sensitivity test was negative, pulp vitality was observed in the main canal and necrosis in the canal related to the dens invaginatus. Some authors argue that endodontic treatment of the main canal is not necessary if there is no connection to the invaginated canal or when necrosis of the pulp is not detected [9, 12]. However, due to the anatomical proximity of the canal entrances and the risk of contamination of the vital canal during the operative event, the endodontic treatment of both canals was performed.

In both cases, the irrigation solution used to aid disinfection of the root canals was 2.5% sodium hypochlorite. The solvent and bacterial properties of this solution, together with the prolonged action of intracanal calcium hydroxide medication were adequate to complement the disinfection of the canals. For most authors, the irrigating solution of choice is sodium hypochlorite at concentrations ranging from 0.5% to 5.25% and the intracanal medication of choice is calcium hydroxide because it stimulates the deposition of mineralized tissue [7, 11]. 

In the reported cases, conventional endodontic therapy was successful and sufficient enough to eliminate the infectious processes of the root canal system, allowing periapical bone neoformation and absence of symptomatology. Dens invaginatus is a relatively easy-to-diagnose dental malformation; however, it is necessary to know its radiographic aspects.

## Conclusion

The results demonstrated that, although the cases of dens invaginatus of high complexity are challenging, an accurate diagnosis associated with proper endodontic treatment can avoid unnecessary surgical intervention and allow excellent chances of a favorable prognosis in the long term.
